# Imaging Modality and Outcomes of Metastasis-Directed Therapy in Oligometastatic Castration-Resistant Prostate Cancer: An Updated Analysis of the PRECISE-MDT Cohort

**DOI:** 10.2967/jnumed.125.271680

**Published:** 2026-09

**Authors:** Matteo Bauckneht, Liliana Belgioia, Domenico Albano, Luca Triggiani, Flavia Linguanti, Luca Urso, Rosario Mazzola, Alessio Rizzo, Elisa D’Angelo, Francesco Dondi, Eneida Mataj, Gloria Pedersoli, Elisabetta Maria Abenavoli, Luca Vaggelli, Beatrice Detti, Naima Ortolan, Antonio Malorgio, Alessia Guarneri, Federico Garrou, Jacopo Passoni, Matilde Fiorini, Serena Grimaldi, Pietro Ghedini, Giuseppe Carlo Iorio, Antonella Iudicello, Guido Rovera, Giuseppe Fornarini, Elisa Zanardi, Diego Bongiovanni, Michela Marcenaro, Alessandra Agosti, Giorgia Timon, Matteo Salgarello, Manuela Racca, Mirco Bartolomei, Stefano Panareo, Umberto Ricardi, Francesco Bertagna, Filippo Alongi, Salvina Barra, Silvia Morbelli, Francesco Lanfranchi, Gianmario Sambuceti

**Affiliations:** 1 IRCCS Azienda Ospedaliera Metropolitana, Genoa, Italy;; 2 Department of Health Sciences, University of Genova, Genova, Italy;; 3 Department of Nuclear Medicine, ASST Spedali Civili di Brescia, Brescia, Italy;; 4 University of Brescia, Brescia, Italy;; 5 Department of Radiotherapy, ASST Spedali Civili di Brescia, Brescia, Italy;; 6 Department of Nuclear Medicine, Ospedale San Donato, Arezzo, Italy;; 7 Department of Nuclear Medicine, University of Ferrara, Ferrara, Italy;; 8 Department of Radiotherapy, Humanitas Gavazzeni Hospital, Bergamo, Italy;; 9 Department of Biomedical Sciences, Humanitas University, Milan, Italy;; 10 Department of Nuclear Medicine, Candiolo Cancer Institute, FPO-IRCCS, Turin, Italy;; 11 Department of Radiotherapy, AUSL of Bologna, Bologna, Italy;; 12 Department of Nuclear Medicine, Careggi University Hospital, Florence, Italy;; 13 Department of Radiotherapy, Azienda USL Centro Toscana, Prato, Italy;; 14 Department of Radiotherapy, University of Ferrara, Ferrara, Italy;; 15 Department of Radiotherapy, Candiolo Cancer Institute, FPO-IRCCS, Turin, Italy;; 16 Department of Nuclear Medicine, ASST-Ospedale Papa Giovanni XXIII, Bergamo, Italy;; 17 Department of Advanced Radiation Oncology, IRCCS Sacro Cuore Don Calabria Hospital, Negrar, Italy;; 18 Department of Nuclear Medicine, AOU Città della Salute e della Scienza di Torino, University of Turin, Turin, Italy;; 19 Nuclear Medicine, Oncology, and Haematology Department, University of Modena, Modena, Italy;; 20 Department of Radiation Oncology, University of Turin, Turin, Italy;; 21 Department of Nuclear Medicine, IRCCS Ospedale Sacro Cuore Don Calabria, Negrar, Italy;; 22 Nuclear Medicine Unit, A.O.U. San Luigi Gonzaga, Turin, Italy; and; 23 Department of Experimental Medicine, University of Genova, Genova, Italy

**Keywords:** prostate cancer, castration-resistant, oligometastatic, PSMA PET/CT, stereotactic radiotherapy

## Abstract

It is unclear whether the imaging modality used to guide metastasis-directed therapy (MDT) influences outcomes in oligometastatic castration-resistant prostate cancer (omCRPC). **Methods:** We performed an updated analysis of the PRECISE-MDT cohort, adding 27 patients to the original cohort. MDT guided by prostate-specific membrane antigen (PSMA) PET/CT, choline PET/CT, and conventional imaging was compared in 102 patients with omCRPC. The primary outcomes were biochemical recurrence–free survival and overall survival after adjustment for inverse probability of treatment weighting. **Results:** MDT was guided by conventional imaging in 20 (19.6%) patients, choline PET/CT in 56 (54.9%) patients, and PSMA PET/CT in 26 (25.5%) patients. PSMA PET/CT guidance yielded longer biochemical recurrence–free survival than did choline PET/CT (median not reached [NR] vs. 11.7 mo, *P* = 0.031) and conventional imaging (NR vs. 7.1 mo, *P* = 0.002). Overall survival favored PSMA PET/CT versus conventional imaging (NR vs. 46.0 mo, *P* = 0.036). **Conclusion:** PSMA PET/CT guidance was associated with improved clinical outcome, supporting its therapeutic relevance for MDT planning in omCRPC.

## Visual Abstract

**Figure:**
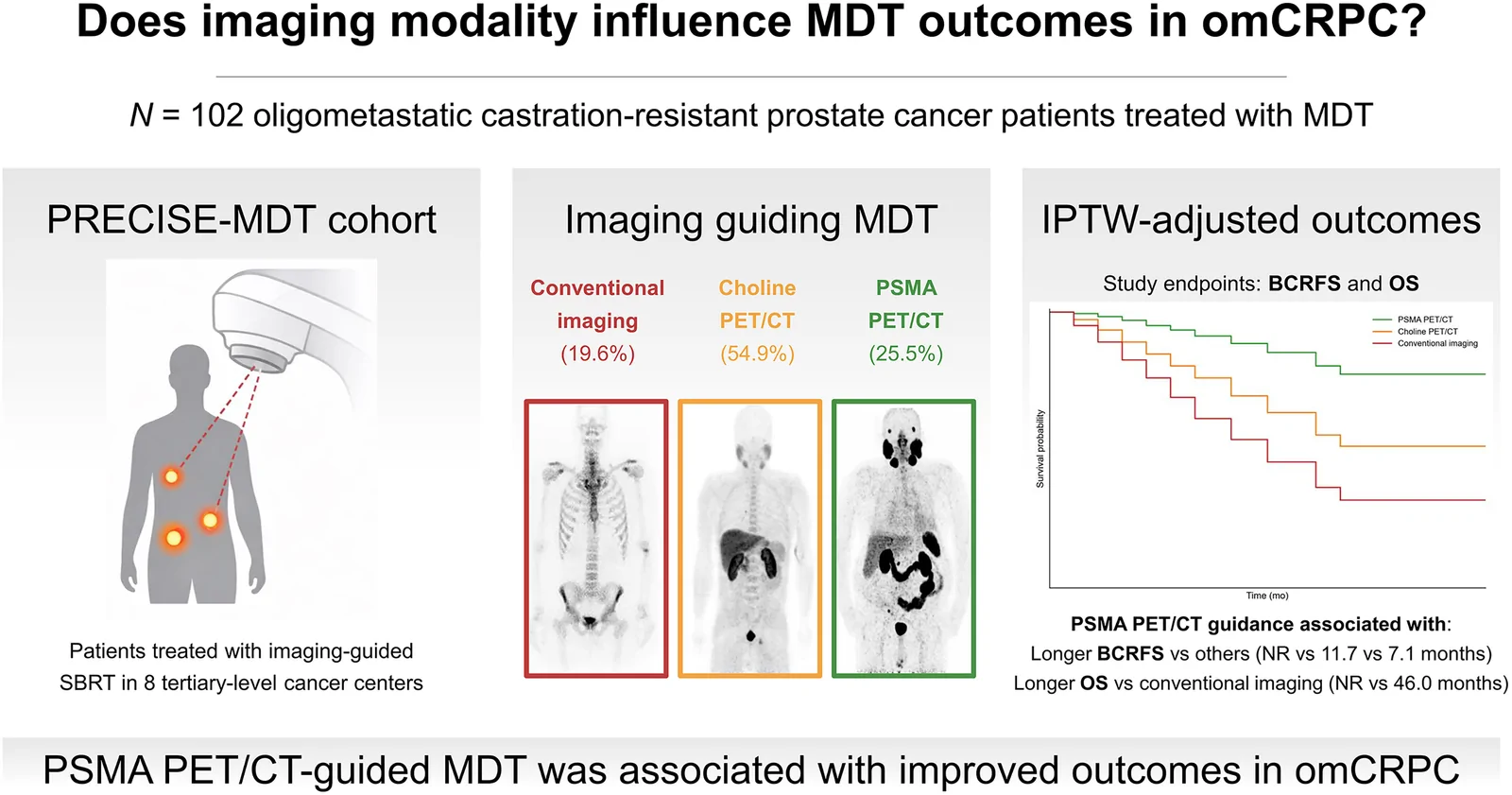

Metastasis-directed therapy (MDT) using stereotactic body radiation therapy offers disease control in oligometastatic prostate cancer (1–6). However, the imaging modalities guiding the MDT vary widely, ranging from conventional imaging to PET/CT, potentially affecting treatment outcomes.

The PRECISE-MDT study, which included patients with both hormone-sensitive prostate cancer and castration-resistant prostate cancer (CRPC), demonstrated improved results with prostate-specific membrane antigen (PSMA) PET/CT–guided MDT compared with choline PET/CT in patients with oligorecurrent prostate cancer (7,8). However, prior analyses have not specifically focused on the oligometastatic castration-resistant prostate cancer (omCRPC) subgroup, and data in this setting remain limited, whereas conventional imaging is still used in clinical practice and trials.

This updated analysis aimed to evaluate the impact of imaging modality (PSMA PET/CT, choline PET/CT, and conventional imaging) on clinical outcomes after MDT in patients with omCRPC, expanding the PRECISE-MDT cohort with additional patients with the disease.

## MATERIALS AND METHODS

### Study Design, Procedures, and Follow-Up

Patients with omCRPC who underwent MDT with stereotactic body radiation therapy were identified from the PRECISE-MDT study cohort (7). CRPC was defined according to European Association of Urology guidelines (9). In the original PRECISE-MDT analysis (including hormone-sensitive disease and CRPC), MDT guided by conventional imaging was excluded from the comparative analysis because all such patients had CRPC, resulting in an imbalance across imaging modalities in the overall cohort. In this CRPC-focused study, these patients were included to enable evaluation of all imaging strategies within a homogeneous disease population. In addition, 7 patients with CRPC who underwent PET-guided MDT during the same study period, but lacked sufficient follow-up for inclusion in the original analysis, were included after follow-up maturation (Supplemental Fig. 1; supplemental materials are available at http://jnm.snmjournals.org).

The study adhered to the Declaration of Helsinki and was approved by the local ethical committee (registration number 5/2023 - DB id 12914). All patients provided written informed consent.

MDT guided by [^18^F]F-fluorocholine, [^68^Ga]Ga-PSMA-11, or [^18^F]F-PSMA-1007 PET/CT was performed in accordance with international guidelines (9,10). Details of the PET/CT scanners used at each center are provided in Supplemental Table 1. MDT was delivered following international recommendations and good clinical practice (11).

Follow-up consisted of clinical evaluation and prostate-specific antigen (PSA) testing every 3–6 mo, beginning at the completion of MDT.

### Statistical Analysis

The primary endpoints were biochemical recurrence–free survival (BCRFS) and overall survival (OS). Secondary endpoints included metastasis-free survival, progression-free survival (PFS), and time to systemic therapy change. BCRFS was defined by the Phoenix criteria (12). Propensity score estimation included the year of diagnosis, International Society of Urological Pathology grade, year of imaging, systemic therapy at the time of MDT, PSA level, and number and site of metastases. Analyses were adjusted using inverse probability of treatment weighting (IPTW) (supplemental materials). Covariate balance was assessed via standardized mean differences. Kaplan–Meier and Cox models were used to compare outcomes across imaging modalities.

## RESULTS

### Cohort Characteristics

In total, 102 patients with omCRPC were included in the present analysis. MDT was guided by conventional imaging, choline PET/CT, and PSMA PET/CT in 20 (19.6%), 56 (54.9%), and 26 (25.5%) cases, respectively. Supplemental Table 2 displays baseline clinical, imaging, treatment, and follow-up characteristics.

At the time of MDT, most patients were receiving androgen deprivation therapy (ADT) alone—60.0%, 76.8%, and 69.2% in the conventional imaging, choline PET/CT, and PSMA PET/CT groups, respectively. ADT combined with androgen receptor signaling inhibitors (ARSIs) was the second most common regimen (35.0%, 17.9%, and 19.2%), followed by ADT plus chemotherapy (5.0%, 5.4%, and 11.5%).

Although not statistically significant, bone metastases were more frequent in the conventional imaging group (60.0%), whereas nodal metastases predominated in the choline and PSMA PET/CT groups (62.5% and 57.7%, respectively). Radiation doses and fractionation schedules were comparable across groups. PSA nadir and PSA at biochemical recurrence did not differ significantly. In most cases, restaging after MDT was performed with the same imaging modality used for treatment guidance.

For all IPTW covariates, standardized mean differences were calculated before and after weighting (Supplemental Table 3; Supplemental Fig. 2).

### BCRFS

**FIGURE 1.**
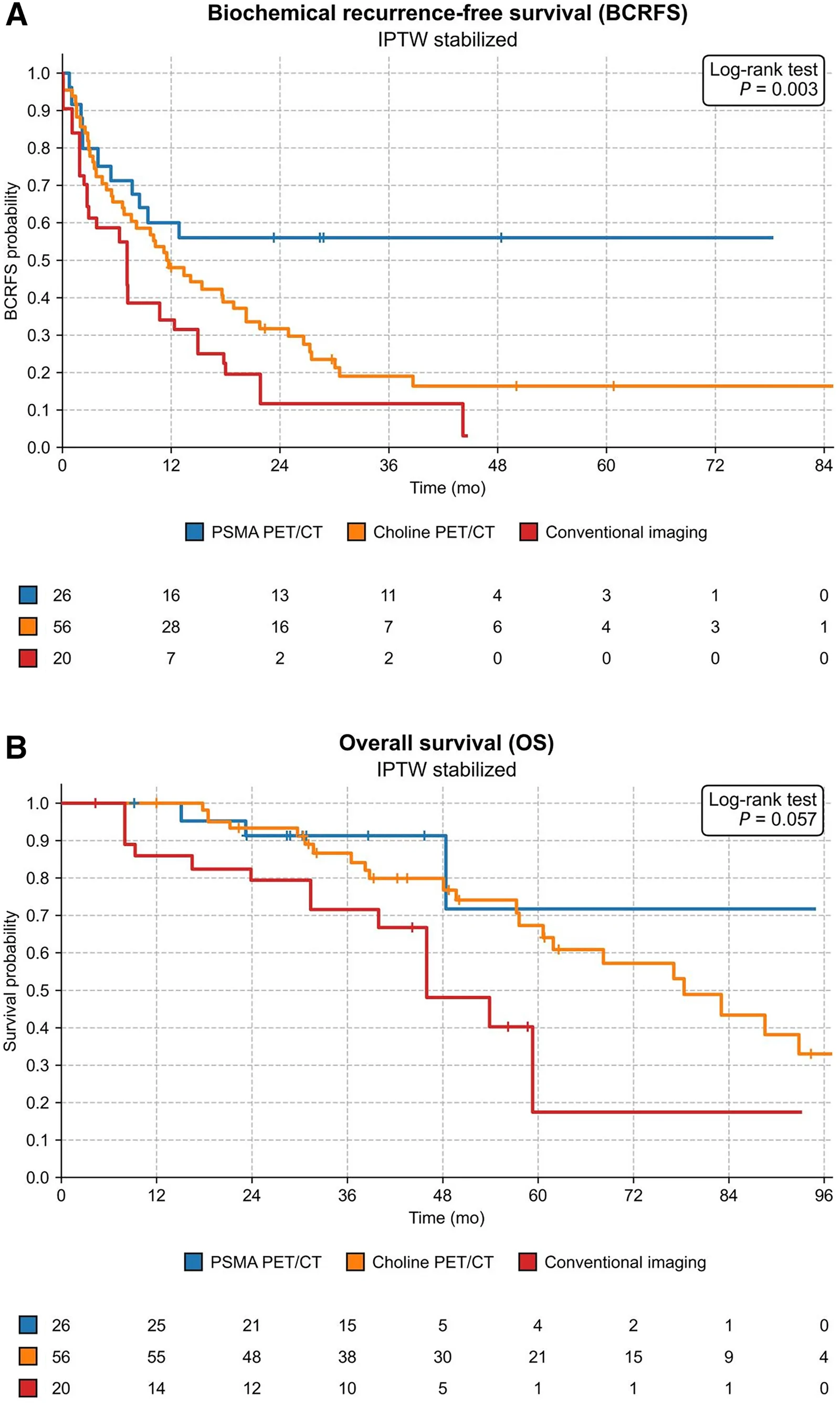
IPTW-adjusted Kaplan–Meier curves for BCRFS (A) and OS (B).

During follow-up, biochemical recurrence occurred in 19 (95%), 44 (78.6%), and 11 (42.3%) patients in the conventional imaging, choline PET/CT, and PSMA PET/CT groups, respectively. PSMA PET/CT guidance (median not reached in PSMA group) was significantly associated with longer BCRFS compared with both choline PET/CT (11.7 mo, *P* = 0.031) and conventional imaging (7.1 mo, *P* = 0.002) (Fig. 1; Supplemental Table 4). These results were consistent in the IPTW-trimmed sensitivity analysis (Supplemental Fig. 3).

### OS

At the time of analysis, 36 deaths were recorded: 10 (50.0%) in the conventional imaging group, 23 (41.1%) in the choline PET/CT group, and 3 (11.5%) in the PSMA PET/CT group. Patients treated with PSMA PET/CT–guided MDT experienced significantly prolonged OS compared with those whose MDT was guided by conventional imaging (median not reached vs. 45.97 mo, *P* = 0.036) (Fig. 1; Supplemental Table 4). The difference between PSMA PET/CT and choline PET/CT did not reach statistical significance (*P* = 0.065). The results remained consistent in the IPTW-trimmed sensitivity analysis (Supplemental Fig. 3).

### Other Clinical Outcomes

New metastatic lesions were detected in 90.0%, 69.6%, and 69.2% of patients in the conventional imaging, choline PET/CT, and PSMA PET/CT groups, respectively. Polymetastatic conversion requiring systemic therapy modification occurred in 14 (70.0%), 35 (62.5%), and 18 (69.2%) cases, whereas disease progression occurred in 100%, 91.1%, and 84.6% of patients, respectively. No significant differences were observed among groups for metastasis-free survival (median, 8.2 vs. 21.0 vs. 11.4 mo), time to systemic therapy change (median, 11.0 vs. 27.6 vs. 12.7 mo), or composite PFS (median, 7.1 vs. 10.6 vs. 7.9 mo) (all *P* > 0.05) (Supplemental Table 4; Supplemental Fig. 4).

## DISCUSSION

While the efficacy of MDT is established in hormone-sensitive disease, its role in the castration-resistant setting remains unclear. CRPC represents a biologically distinct phase, characterized by increased tumor heterogeneity and potential alterations in PSMA expression that may influence the performance of PSMA PET/CT for MDT planning. Therefore, the clinical relevance of PSMA PET/CT–guided MDT in this setting requires dedicated evaluation. In this updated secondary analysis of PRECISE-MDT, PSMA PET/CT–guided MDT was associated with longer biochemical control and OS compared with other imaging modalities. Whether this association reflects improved patient selection, enhanced lesion detection and targeting, or residual confounding cannot be determined from this analysis. Moreover, given the retrospective design, these findings should be interpreted as associative rather than causal.

These results align with and expand recent evidence. The ARTO phase 2 trial demonstrated that combining MDT with ARSIs significantly improved PFS compared with ARSIs alone in patients with omCRPC (4). A subsequent ARTO subgroup analysis found that, after progression on first-line ARSIs, MDT for oligoprogression yielded PFS and OS comparable to those of second-line systemic therapy, suggesting that MDT can defer escalation in selected patients (13). Similarly, the MEDCARE single-arm prospective phase 2 study found longer failure-free survival when all PSMA-positive lesions were treated, rather than leaving residual PSMA-positive disease undetected by conventional imaging, although the sample size was limited (14). These data indicate that both imaging sensitivity and ablation completeness are key determinants of MDT efficacy in the castration-resistant setting. Conversely, the definition of heterogeneous disease burden across the predominantly retrospective literature (15) varies, making the efficacy of MDT difficult to compare (16).

This updated analysis addresses a limitation of the original PRECISE-MDT study (7), which lacked power for a dedicated CRPC analysis. By including additional patients with omCRPC, this analysis provides a more comprehensive evaluation of imaging-guided MDT within this specific disease setting. Although causality cannot be proven, the consistent survival advantage for PSMA PET/CT in adjusted analyses supports a meaningful association between advanced imaging and improved outcomes.

This study had several limitations. The retrospective, observational design may have introduced selection bias (including survivorship) and limited statistical power. Although IPTW was applied to balance key prognostic variables, residual confounding cannot be excluded. In particular, heterogeneity in systemic therapy and restaging schedules may have influenced results, and detailed data on the types, duration, and sequence of prior systemic treatments were not consistently available and therefore could not be modeled explicitly. These factors preclude firm causal inferences linking imaging modality to clinical benefit. Nevertheless, this study has notable strengths. It represents an updated, multicenter, real-world analysis; uses rigorous propensity-based weighting; and reflects contemporary clinical practice, thereby enhancing generalizability.

## CONCLUSION

This updated PRECISE-MDT analysis suggests that PSMA PET/CT–guided MDT is associated with improved outcomes compared with other imaging modalities in omCRPC. Given the retrospective design, it remains unclear whether these findings reflect better patient selection, more accurate lesion targeting, or residual confounding. Prospective validation of these results is warranted.

## DISCLOSURE

Matteo Bauckneht reports personal fees from Novartis, Bayer, Telix Pharmaceuticals, Recordati, and Johnson & Johnson. Elisa Zanardi reports personal fees from Astellas, Ipsen, and Johnson & Johnson. No other potential conflict of interest relevant to this article was reported.

## Notes

KEY POINTS **QUESTION:** Does imaging modality influence MDT outcomes in patients with omCRPC? **PERTINENT FINDINGS:** In a multicenter IPTW-adjusted cohort of 102 patients, PSMA PET/CT–guided MDT was associated with longer biochemical control and OS compared with other imaging modalities. **IMPLICATIONS FOR PATIENT CARE:** PSMA PET/CT guidance was associated with improved clinical outcomes, supporting its therapeutic relevance for MDT planning in omCRPC.
